# Tiger-like late gadolinium enhancement pattern in aborted myocardial infarction

**DOI:** 10.1093/ehjcr/ytaf642

**Published:** 2025-12-26

**Authors:** Salome Tsintsadze, Thomas de Beenhouwer, Panagiotis Xaplanteris, Mihaela Silvia Amzulescu

**Affiliations:** Tbilisi Institute of Medicine, Sulkhan Tsintsadze Street 16, Tbilisi 0160, Georgia; CHU Saint Pierre, Rue aux Laines 105, Brussels 1000, Belgium; CHU Saint Pierre, Rue aux Laines 105, Brussels 1000, Belgium; CHU Saint Pierre, Rue aux Laines 105, Brussels 1000, Belgium

A 50-year-old male with a history of hypertension and active smoking presented to the emergency room complaining of long-lasting, intermittent chest pain, radiating to the jaw and shoulders. ECG showed anteroseptal Q waves and antero-lateral T-wave inversion (*Figure 1A*). Peak troponin T level was 2000ng/L. On echocardiography, the anteroseptal wall was hypokinetic. The coronary angiography revealed an intermediate lesion of the mid-segment of the LAD artery, estimated at 50% (*Figure 1C*). No percutaneous coronary intervention (PCI) was performed, and assessment by fractional flow reserve (FFR) was proposed in the follow-up.

**Figure 1:**
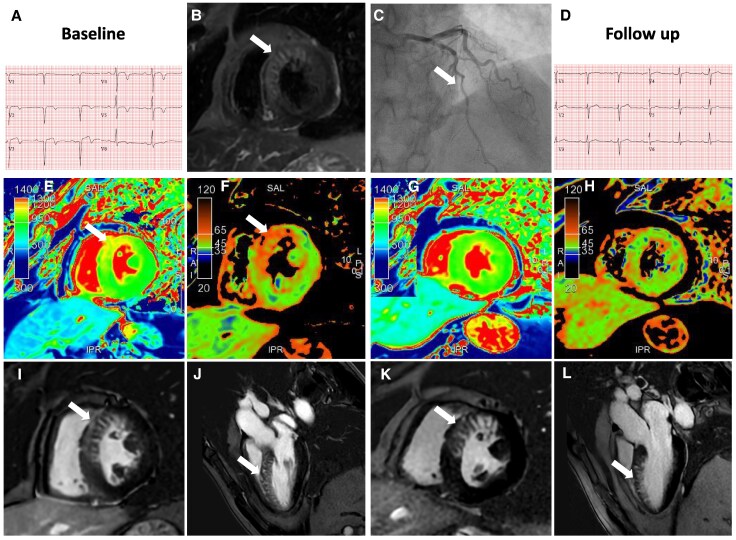
Left (baseline) and right (follow-up) panel. Electrocardiogram showing anteroseptal Q waves and antero-lateral T wave inversion at baseline (*A*), normalized at 6-month follow-up (*D*). Coronary angiography showing a 50% lesion in the mid-segment of the LAD (*C*, arrow). Cardiac MRI black-blood short inversion time recovery (STIR) images showing focal myocardial oedema in the mid anteroseptal region (*B*, arrow). T1 and T2 mapping showing diffuse myocardial oedema at baseline (*E* and *F*, arrow), resolved at follow-up (*G* and *H*). Phase-sensitive inversion recovery (PSIR) late gadolinium enhancement (LGE) sequences showing the presence of a particular patchy, tiger pattern (arrows) in the territory of the mid LAD at both baseline (*I* and *J*) and follow-up (*K* and *L*).

The 1.5-T CMR showed left ventriclular (LV) hypertrophy (LV mass 135 g) and mildly reduced function [ejection fraction (EF) 48%] (see Supplementary material online, *Video S1*). The black-blood short inversion time recovery (STIR) images showed focal myocardial oedema in the mid anteroseptal region (*Figure 1B*), while T2 mapping additionally showed more extended diffuse oedema (*Figure 1F*). The native T1 values were increased in the basal anterior and anteroseptal region (*Figure 1E*). The Phase-sensitive inversion recovery (PSIR) sequences showed the presence of a particular patchy pattern of LGE in the territory of the mid LAD (*Figure 1I* and *J*). Notably, the LGE was distributed as transversal septal stripes interposed in-between areas of non-enhanced myocardium, giving the appearance of a tiger-like LGE pattern. At the 6-month follow-up, the ECG improved and CMR showed the regression of the myocardial oedema (*Figure 1G* and *H*), but persistent tiger-like transmural pattern (*Figure 1K* and *L*).

This aspect is a novel pattern that has not been yet described as associated with spontaneously aborted myocardial infarction. The exact reason for such pattern is not known, although we can speculate on a possible reperfusion injury, although the disposition is atypical. This diagnostic hypothesis is sustained by the clinical presentation, cardiovascular risk factors, elevated troponin levels, ECG changes and LGE distribution matching the LAD territory. The differential diagnosis could consider zebra-like metal artefacts due to magnetic field inhomogeneity,^1^ several small coronary fistulas or multiple myocardial crypts.^2^

## Supplementary Material

ytaf642_Supplementary_Data

## Data Availability

The data underlying this case will be shared on reasonable request to the corresponding author.

## Notes

**Consent:** Informed consent was obtained from the patient for publication of this case. This case report has been prepared in accordance with the ethical standards of the Declaration of Helsinki and the Committee on Publication Ethics (COPE) guidelines.
